# Molecular Detection and Analysis of *Trypanosoma* (*Megatrypanum*) spp. Diversity in Tabanidae (Diptera) Collected in Lithuania

**DOI:** 10.3390/insects15080581

**Published:** 2024-07-30

**Authors:** Jurga Turčinavičienė, Rasa Bernotienė, Andrius Petrašiūnas

**Affiliations:** Department of Zoology, Institute of Biosciences, Life Sciences Center of Vilnius University, Saulėtekio Av. 7, LT-10257 Vilnius, Lithuania; rasa.bernotiene@gamtc.lt

**Keywords:** *Tabanus*, *Hybomitra*, *Haematopota*, *Trypanosoma theileri*, *Megatrypanum*, Lithuania

## Abstract

**Simple Summary:**

Horse flies are known as vectors of various species of trypanosomatids. The aim of this study was to investigate *Trypanosoma* in the Tabanidae family collected in different localities in Lithuania. Our objectives were to examine the genetic diversity of *Trypanosoma* sp. detected in tabanids, assessing the relationships between parasite lineages and tabanid species. The present study reports on the molecular detection and genetic diversity of trypanosomes in 10 species of tabanids, with two of them being reported for the first time. The findings suggest that different strains of the *T. theileri* complex are not related to different tabanid species.

**Abstract:**

Trypanosomatids from the Tabanidae family have not been studied in Lithuania in any detail. In this study, a nested PCR amplifying the DNA fragment coding the SSU rRNA was used to determine the *Trypanosoma* spp. prevalence and diversity in the Tabanidae family collected in Lithuania in 2018–2019. In total, 101 Tabanidae individuals were investigated from six areas in Lithuania, and 14 different species were identified. The overall positivity of *Trypanosoma* spp. DNA in tabanids was 50.5% (51/101). *Tabanus maculicornis* was the most abundant species and yielded the highest prevalence of trypanosomatids (84.62%, 22/26), while *Hybomitra nitidifrons* showed a high prevalence as well, reaching 77.8% (14/18). In flies of some species (*Hybomitra lapponica* and *Hybomitra lurida*), *Trypanosoma* was detected for the first time. Nine different haplotypes were detected as being distributed in different tabanid species. Analysis showed that most sequences obtained during our study were identical or extremely close to two major *T. theileri* subclades: TthI and TthII. Our data analysis suggests the presence of different *Trypanosoma* genotypes in the same tabanid species, meaning that different lineages of *Trypanosoma* could be more related to the vertebrate host and not the fly species. This is the first study of trypanosomatid parasites in tabanids from Lithuania, and our results are valuable in providing data on the diversity of these parasites in different Tabanidae species.

## 1. Introduction

The family Tabanidae (Diptera) consists of haematophagous insects which, due to their lifestyle and feeding behavior, play a role as vectors in the mechanical and biological transmission of a large variety of disease agents. However, distinct species of Tabanidae can act as vectors of different pathogens.

Thirty-nine Tabanidae (horsefly and deer fly) species have been detected thus far in Lithuania [1], although there are no data regarding the abundance of each species in the country. Tabanids are known as biological and mechanical vectors of viruses, such as equine infectious anemia and hog cholera, bacteria such as *Bacillus anthracis*, *Francisella tularensis* and *Anaplasma marginale*, protozoans such as *Trypanosoma* and *Besnoitia* and filarial nematodes [2]. The aim of this study was to determine the prevalence of *Trypanosoma* spp. and the diversity in the Tabanidae members collected in Lithuania.

Tabanids are known to mechanically transmit different trypanosomatids, such as *Trypanosoma* (*Trypanozoon*) *evansi* (Steel, 1885) Balbiani, 1888, the causal agent surra of camels, horses and other mammals. Tabanids are also known to mechanically transmit *T.* (*Duttonella*) *vivax* Ziemann, 1905 (South America) and *T.* (*Trypanozoon*) *brucei* Plimmer and Bradford, 1899 (parts of Africa) [2]. Tabanids are considered vectors of the *Trypanosoma* (*Megatrypanum*) *theileri* Laveran, 1902 species complex [3,4] with the species *T. theileri.* The pathogen can be transmitted by laying infected feces on the skin of the mammalian host or through ingestion of infected insects [5]. The T. theileri species complex consists of several species (T. theileri, T. melophagium, T. cervi, *T*. cf. *cervi* and T. trinaperronei) and various trypanosome genotypes and lineages reported from cervids, bovids and insects, but the exact taxonomy of these is not yet possible to determine [6,7,8]. The status of trypanosomatid species remains unclear, as it does for all organisms with predominantly clonal reproduction [9], and the taxonomy of the *T. theileri* species complex is still controversial. All trypanosomes of this group have unique *T. theileri*-like morphology and are the largest mammalian blood trypanosomes with small kinetoplasts. Based on their morphology, they were merged into the subgenus *Megatrypanum* and classified according to the hosts they infect as well. The most common hosts are domestic and wild ruminants [10]. The phylogeny based on the SSU rRNA of the *T. theileri* group showed a strongly supported and monophyletic clade [11] which, despite high similarity, was separated from other *T.* (*Megatrypanum*) trypanosomes (*T. conorhini*, *T. legeri*, *T. minasense*, *T*. *pestanai* and *T. binneyi*). Rodrigues et al. [11] indicated that the name *T*. (*Megatrypanum*) should only apply to members of the *T. theileri* clade and not to other species.

*Megatrypanum*, found in bovids and cervids, are transmitted by tabanid flies, whereas those infecting sheep and goats are transmitted by host-specific louse flies (Hippoboscidae). Interrupted feeding could be the most important factor determining the role of tabanids as effective mechanical vectors of various pathogens [12]. *Trypanosoma* (*Megatrypanum*) are commonly found in the hindgut of tabanids but are absent from the salivary glands, meaning that the transmission occurs primarily through feces entering the bite wound or perhaps the ingestion of infected tabanids by the animal [5]. The high degree of host specificity, evidenced by genotypes exclusive to each ruminant species and the lack of a genotype shared by different host species, suggest that the evolutionary history of trypanosomes of this subgenus is strongly constrained by their ruminant hosts [11]. Recent phylogenetic studies segregated *T. theileri* in cattle and other ruminants worldwide into two major genetic lineages (the TthI and TthII clades) based on genetic markers [13,14].

Species of the *T. theileri* complex are distributed in many regions, but only few investigations confirmed their occurrence in tabanids. Trypanosomatid infections (*T. theileri*-like) in four species of female horseflies (*Hyb. tarandina*, *Hyb. muehlfeldi*, *Hyb. bimaculata* and *Chrysops divaricatus*) were detected in northwest Russia [15]. Detection of *Trypanosoma* (*Megatrypanum*) in *Haematopota pluvialis*, *T. bromius*, *T. maculicornis* and *T. distinguendus* was reported in Poland [16], and *Hyb. ciureai* and *T. bromius* have been found to be positive for *T. theileri* in the Czech Republic [6]. In one study, fifty-five percent of host-seeking *Chrysops* in North America were infected with trypanosomatid parasites [17]. The infective stages of *T. theileri* were identified using transmission experiments [5], and the development stages of two species of the *T. theileri* complex in tabanids were described [18].

Tabanids in Lithuania were not studied earlier for the presence of trypanosomatids. We collected tabanids from 2018 to 2019 at several sampling localities in Lithuania in order to find which species of trypanosomatids are present in the area and to determine their prevalence in tabanids of different species.

The objectives of this study were (1) to collect tabanids in different localities in Lithuania and to investigate them for the presence of *Trypanosoma* and (2) to examine the genetic diversity of the detected *Trypanosoma* sp. and to assess the relationships between the parasite lineages and tabanid host species. To address these questions, we evaluated the genetic diversity of *Trypanosoma* isolates from 14 different species of Tabanidae. The knowledge of pathogens, which can be detected in insects of particular species, would give new insights into insect feeding preferences as well as potential vector species and would allow better understanding of the transmission regularities of pathogens in the wild.

## 2. Material and Methods

### 2.1. Tabanid Fly Collection and Identification

Tabanid specimens were collected with Nzi traps [19] in four localities in Lithuania—Azuolu Buda (N54.70761, E23.53323), Ilgakiemis (N54.76593, E23.83295), Poskaiciai (N54.89185, E25.42979), Sirvydai (N55.00955, E23.45876)—and with an entomological net in two additional localities—Papile (N56.17059, E22.78323) and Sviliai (N55.26670, E23.79309)—from June of 2018 to September of 2019.

Species identification was carried out using a taxonomic key [20] before processing. Morphological identification of flies with uncertain identification was confirmed using PCR. Fragments of the cytochrome oxidase I gene were amplified using universal primers LCO1490 and HCO2198. They were then sequenced, and the identities of the obtained sequences were checked using the “Basic Local Alignment Search Tool” (National Centre of Biotechnology Information website: http//www.ncbi.nlm.nih.gov/BLAST (accessed on 1 May 2024). The obtained sequences (accession numbers PP946791–P946795) matched the sequences from GenBank more than 99% of the time. Voucher specimens were deposited at Vilnius University’s Department of Zoology.

### 2.2. The Detection of Trypanosoma in Tabanid Flies Using PCR

The total DNA was extracted from each individual insect using a DNeasy Blood&Tissue Kit (QIAGEN, Hilden, Germany) according to the manufacturer’s instructions. A nested PCR protocol was applied to amplify a 748 bp-long DNA fragment encoding SSU rRNA using the outer primers Tryp763 and Tryp 1016 and the inner primers Tryp99 and Tryp957 [21]. All PCRs were performed in a total volume of 25 µL: 2 µL total genomic DNA template, 12.5 µL DreamTaq Master Mix (Thermo Fisher Scientific, Lithuania, Vilnius), 8.5 µL nuclease-free water and 1 µL of each primer. The temperature profiles in all PCRs were the same, as provided by Sehgal et al. [21]. All amplifications were evaluated using electrophoresis. One positive control (*Trypanosoma* sp. infected insect, confirmed by sequence analysis) and one negative control (ultrapure water) were used during each amplification.

### 2.3. Sequence Analysis

The PCR-positive samples were purified and sequenced using a Big Dye Terminator V3.1 Cycle Sequencing kit and ABI PRISM^TM^ 3100 capillary sequencing robot (Applied Biosystems, Foster City, CA, USA), and the *Trypanosoma* sequences were identified using the “Basic Local Alignment Search Tool” available from the NCBI. The sequences were submitted to GenBank (GenBank accession no. PP945809-PP945817). Other sequences for the *Megatrypanum* trypanosomes included in the phylogenetic analyses were obtained from GenBank (Table 1). The newly generated sequences were aligned with other *Megatrypanum* trypanosomes which belonged to the TthI and TthII clades of *Trypanosoma theileri*.

To clarify the intra-species relationships among the *T. theileri* species group, a phylogenetic median-joining (MJ) network [22] was created using Popart-1.7 software (http://popart.otago.ac.nz, accessed on 17 February 2023 [23]). This method, described in detail in [22], includes nodes to represent inferred sequences by iteratively adding “median” sequence vectors, and it combines the features of Kruskal’s algorithm. The MJ method begins with the minimum spanning trees, all combined within a single network while sequentially adding a few consensus sequences of three mutually close sequences at a time.

**Table 1:** List of species where trypanosomes were detected and sequence numbers from the GenBank database used in this study. Sequences in bold (GenBank accession nos. PP945809–PP945817) were determined in the present study. Numbers in network as shown in Figure 1.

Number in Network (Haplotype)	Species and Specimen ID	Number of Identical *Trypanosoma* spp. Sequences	GenBank Accession No.	Species According to Data in GenBank and References
1	*Haematopota pluvialis* 113	1	**PP945809**	
	*Haematopota subcylindrica* 310	1		
	*Hybomitra distinquenda* 215	1		
	*Hybomitra muehlfeldi* 425	1		
	*Hybomitra nitidifrons* 418, 428	2		
	*Tabanus bromius* 458, 511, 513	3		
	*Tabanus maculicornis* 512, 514, 519, 520, 524, 527, 528	7		
	*Coquillettidia richiardii*	1	OM256570	*T. theileri* II (complex II [6])
	*Cervus nippon*	1	LC618030	*T. theileri* II (complex II [13])
2	*Hybomitra muehlfeldi*	1	OL855998	*T. theileri* (complex II [18])
	*Hybomitra tarandina*	1	MK156791	*T. theileri* II (complex II [7])
	*Tabanus maculicornis* 523	1	**PP945810**	
3	*Odocoileus virginianus*	1	JX178196	*Trypanosoma* cf. *cervi* ([24])
	*Tabanus maculicornis* 532	1	**PP945811**	
4	*Hybomitra lurida* 37	1	**PP945812**	
	*Hybomitra nitidifrons* 420, 426	2		
	*Chrysops divaricatus*	1	MK156793	*T. theileri* I (complex I [15])
	*Glossina fuscipes fuscipes*	1	KR024688	*T. theileri* ([25])
	*Hybomitra muehlfeldi*	1	MK156792	*T. theileri* I (complex I [15])
5	*Hybomitra bimaculata* 413, 417	2	**PP945813**	
	*Hybomitra lapponica* 419	1		
	*Tabanus maculicornis* 438	1		
6	*Tabanus bromius* 456	1	**PP945814**	
	*Tabanus maculicornis* 526	1		
7	*Hybomitra nitidifrons* 430	1	**PP945815**	
8	*Haematopota pluvialis* 138	1	**PP945816**	
9	*Tabanus maculicornis* 521	1	**PP945817**	
10	*Melophagus ovinus*	1	HQ664912	*T. melophagium* ([26])
11	*Odocoileus virginianus*	1	MN752212	*T. trinaperronei* ([7])

## 3. Results

### 3.1. Prevalence of Trypanosomatids in Tabanidae

In total, 101 Tabanidae members were investigated from six areas in Lithuania, and 14 different species were identified. Identification of some species (*Hyb. muehlfeldi, Hyb. distinquenda, Hyb. bimaculata, Hyb. nitidifrons* and *T. bromius*) was double-checked using both morphology and barcode sequencing. The most abundant species were *T. maculicornis* and *Hyb. nitidifrons* (Table 2).

In all, 30 newly determined partial SSU rRNA sequences (748 bp) of *Trypanosoma* spp. were obtained. The overall positivity of *Trypanosoma* spp. DNA in the tabanids was 50.5% (51/101) (Table 2), and the DNA of *Trypanosoma* spp. was detected in horseflies of 10 species out of the 14 investigated. *Trypanosoma* DNA was detected in 22 individual *T. maculicornis* specimens out of 26 (84.62%), and lower positivity was observed in *Hyb. nitidifrons* (14/18, 77.8%) and *T. bromius* (6/13, 46.2%). Although flies of other species, such as *Haem. pluvialis* and *Haem. subcylindrica*, were abundant, trypanosomatid DNA was detected only in a few of them (Table 2). Four horsefly species were represented by a single individual each (*Hyb. distinquenda, Hyb. lapponica, Hyb. lurida* and *Hyb. muehlfeldi*) and were positive for *T. theileri.* No trypanosomes were detected in flies belonging to the genus *Chrysops* (*C. italicus, C. relictus* and *C. caecutiens)* or *Haem. crassicornis* during this study, supposedly because of the low number of investigated flies for these species. To the best of our knowledge, the trypanosomatid detections in *Hyb. lurida* and *Hyb. lapponica* are the first ones ever recorded. Five samples from *Hyb. nitidifrons* and one from *T. maculicornis,* positive for *Trypanosoma* spp., carried a mix of either two or more different trypanosomatid haplotypes showing double peaks in the chromatograms of their sequences.

### 3.2. Haplotype Network Analysis

**Figure 1:**
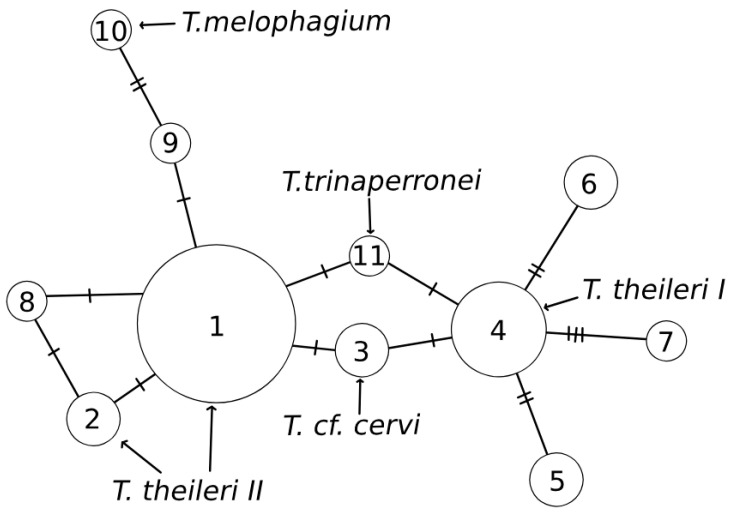
Median-joining network of *Trypanosoma* spp. found in insects (tabanids, culicids and *Glossina fuscipes fuscipes*), *T*. *trinaperronei* found in white-tailed deer and *T. melophagium* inferred using SSU rRNA (748 bp) sequences and median-joining network method for intraspecific phylogenies. Network demonstrates evidence of TthI (circle 4) and TthII (circles 1 and 2) phylogenetic lineages, where most sequences belong. Numbers in circles correspond to those in Table 1.

A phylogenetic median-joining (MJ) network was inferred from 30 newly determined partial SSU rRNA sequences and 8 already-published sequences (Table 1). Sixteen sequences which were isolated from seven tabanid species (seven sequences from *T. maculicornis,* three from *T. bromius,* two from *Hyb. nitidifrons* and one from each specimen of *Haem. pluvialis*, *Hyb. distinquenda, Haem. subcylindrica* and *Hyb. muehlfeldi*) showed 100% identity of the cervid *T. theileri* (GenBank accession no. LC618030, YMG-11) isolated from *Cervus nippon* and *Trypanosoma* sp. isolated from *Coquillettidia richiardii* (Culicidae) (GenBank accession no. OM256570) (Figure 1, circle 1). These two sequences from GenBank belong to bovine *T. theileri* complex II [6,13]. One sequence from *T. maculicornis* had 100% similarity to the *Trypanosoma* sp. sequence isolated from the horsefly *Hyb. tarandina* from northwest Russia (GenBank accession no. MK156791.1, KrS11) [15,18] and from *Hyb. muehlfeldi* (GenBank accession no. OL855998) [18]. Both sequences belong to the *T. theileri* ThII clade (Figure 1, circle 2). A sequence from *Haem. pluvialis* was unique (Figure 1, circle 8), another was 99.9% similar to sequences belonging to TthII (Figure 1, circles 1 and 2), and one sequence from *T. maculicornis* (Figure 1, circle 9) was more closely related to *T. melophagium* (GenBank accession no. HQ664912) [26], a parasite of the sheep *Melophagus ovinus,* with a measured similarity of 99.73%. It was confirmed that *T. melophagium* is more related to the lineage of TthII, and these SSU rRNA sequences are highly conserved among *Megatrypanum* trypanosomes. The differences are only a few substitutions, despite the fact that *T. melophagium* is a different species and has different hosts. Several isolates of *Trypanosoma* from the hosts *Hyb. lurida* and *Hyb*. *nitidifrons* (Figure 1, circle 4) exhibited 100% similarity to the *T. theileri* isolate G24 from *Glossina fuscipes fuscipes* (Central African Republic, GenBank accession no. KR024688) [25] and from the horseflies *Hyb. muehlfeldi* (MK156792), *Chrysops divaricatus* (MK156793) in the Republic of Karelia [15]. Both isolates from these horseflies belong to the complex TthI. Several sequences of *Trypanosoma* isolated from *T. maculicornis* were identical to those referred to as *T*. cf. *cervi* (GenBank accession no. JX178196) from *Odocoileus virginianus* (USA) (Figure 1, circle 3). These isolates occurred between two *T. theileri* complexes in the network (Figure 1) as well as an isolate from the same white-tailed deer, named as a separate species (*T.* (*Megatrypanum*) *trinaperronei*).

## 4. Discussion

Species of the *T.* (*Megatrypanum*) *theileri* group putatively are non-pathogenic mammalian parasites and are transferred by an infected vector. Böse et al. [5] experimentally proved that the transmission of *T. theileri* in the field usually takes place through contamination of the oral mucosa with metacyclic stages from the gut contents or feces of tabanids. Kostygov et al. [18] studied the development of two different species of the *T. theileri* complex in the intestine of the vector and concluded that the observed differences were associated with the species of the vector and not with the phylogenetic position of the parasites. Ganyukova et al. [15] studied four species of infected horseflies and detected four morphotypes of *T. theileri*-like trypanosomes in the hindgut. PCR-based methods are used for detection of these parasites in bloodsucking insects, and Werszko et al. [16] detected *Megatrypanum* trypanosomes in *Haem. pluvialis, T. bromius, T. maculicornis* and *T. distinguendus* in Poland by molecular means, with the overall positivity of *Trypanosoma* spp. being 33.68%. *Trypanosoma theileri* was detected in the tabanids of two species (*Hyb. ciureai* and *T. bromius*) as well as in other bloodsucking Diptera (mosquitoes and sandflies) in the Czech Republic [6], and the prevalence of *T. theileri* in tabanids was reported to be 44%. In Lithuania, only *Trypanosoma* in raptor birds and biting midges have been investigated thus far [27,28], and the subgenus *Megatrypanum* in mammals or tabanids has not been studied yet.

Our study focused on the molecular detection of trypanosomatids in Tabanidae, and we examined a wider range of species and more individuals than in previous studies [6,15,16]. In flies of some species (*Hyb. lapponica* and *Hyb. lurida*), *Trypanosoma* was detected for the first time. *T. maculicornis* was the most abundant species, and this tabanid also yielded the highest prevalence of trypanosomatids (84.62%). Similarly, high prevalence for *Trypanosoma* spp. was detected for this species in Poland (60%) [16]. *Hyb. nitidifrons* showed 77.8% prevalence of *T. theileri* and confirmed the results of a previous study on the development of *T. theileri* in this species of tabanids [18]. Our findings suggest that *T. maculicornis* and *Hyb.nitidifrons* could be effective vectors of *T. theileri*. Some species of tabanids, such as *T. bromius*, have been previously confirmed as vectors of *T. theileri* [5], where 14% of tabanids were positive for trypanosomatids. A high prevalence in *T. bromius* (46.2%) was detected during our study as well as in a study by Brotánková et al. (50%) [6], but this species was not as prevalent (17.95%) for trypanosomatids in Poland [16]. Some abundant tabanid species from our study (*Haem. pluvialis* and *Haem. subcylindrica)* demonstrated low prevalence of *T. theileri*, which is quite surprising because *Haem. pluvialis* showed the highest infection rate (57.14%) in a study carried out in Poland [16].

Five SSU rRNA sequences of *Trypanosoma* spp. obtained from *Hyb. nitidifrons* and one from *T. maculicornis* demonstrated double peaks in their chromatograms, suggesting mixed trypanosomatid infections in the same insect. No infections of *Trypanosoma* spp. were detected in the *Chrysops* flies during our investigation, while the presence of *T. theileri*-like trypanosomatids was reported in these flies in earlier works [5,15]. This observation suggests that larger tabanids such as *Tabanus* or *Hybomitra* transfer more secretion to another host during feeding, possibly influencing the amount of infection of different strains or species of *Trypanosoma* spp., while smaller tabanids, like *Chrysops*, transfer fewer secretions to another host during feeding [12], making these tabanids less important in pathogen transmission.

There is no information concerning the occurrence of *T. theileri* parasites in wild animals in Lithuania, but data available on Polish cervids [29] show the presence of trypanosomes in red deer (*Cervus elaphus*), suggesting that trypanosomes can be found in cervids in Lithuania as well.

*Trypanosoma* sequences from tabanids obtained in the present study showed low genetic polymorphism, and similar to previous studies [11], we detected homogeneity in the *T. theileri* trypanosomes as well. Despite the fact that Schoener et al. [8] reported *T. theileri* as a complex of species that cannot be resolved using the SSU gene, nine different haplotypes distributed in different tabanid species were detected by us (Table 1 and Table 2). It is known that the *T. theileri* group consists of several species (*T. theileri, T. melophagium, T. cervi* and *T. trinaperronei*) [6].

Analysis showed that most sequences obtained during our study were identical or quite close to two major *T. theileri* clades: TthI and TthII (Figure 1, circles 1, 2 and 4). The majority of the sequences were included in the TthII clade (Figure 1 and Table 1) along with a sequence from cervids [13], despite the fact that some authors [7,24] think that complex TthII is genetically closer to bovine tryposomatids and TthI is closer to cervids. The sequence of *T. melophagium* from Croatia (Figure 1, circle 10) was more related to the lineage TthII, as was confirmed by Martinković et al. [26]. The clade TthI contains sequences obtained only from tabanids (Figure 1, network number 4), with only one sequence from *Glossina fuscipes fuscipes* [25]. Some sequences from *T. maculicornis*, *T. bromius*, *Hyb. bimaculata* and *Hyb. lapponica* differed from the sequences available in GenBank and appeared to be closer to the TthI lineage, showing that this group can vary more genetically.

Two different clusters in the constructed network (Figure 1) can be observed. It is worth pointing out that most of the obtained sequences were clustered with other genotypes of two *T. theileri* clades. One sequence from *T. maculicornis* and two isolates from white-tailed deer, described as *T. trinaperronei* n.sp. [7] and *T.* cf. *cervi*, clustered between complexes TthI and TthII, indicating similarity to both clades (Figure 1, circles 3 and 11). Similar results, where the lineage from the deer was recognized as a separate lineage, were demonstrated by Rodrigues et al. [11]. Our network showed close relationships for the trypanosomes of the subgenus *Megatrypanum* and small polymorphism between the lineages.

Confirming previous studies [30], our data analysis suggests the presence of different *Trypanosoma* genotypes in the same tabanid species, meaning that different lineages of *Trypanosoma* could be more related to the vertebrate hosts but not the tabanid species. *Trypanosoma* spp. spread among hosts through tabanids, which feed on blood from a range of species, and thus the existence of several defined genotypes in the same species is probable. The sequences from *T. maculicornis* had six different haplotypes (Table 2), and any degree of host specificity was not found. This tabanid species is abundant and is infected by trypanosomatids more often (84.6%) than other tabanid species.

To the best of our knowledge, this is the first study on *Trypanosoma* genetic diversity in Tabanidae from Lithuania. Although widespread, the *T. theileri* group is largely neglected due to its low economic importance and causing no pathology. However, pathologies might have resulted from coinfections or stress when fever, anorexia, and anemia were reported as symptoms in several bovid infections [31,32].

## 5. Conclusions

The present study reported on the molecular detection and genetic diversity of trypanosomes in 10 species of tabanids, with two of them being reported for the first time. The findings suggest that different strains of the *T. theileri* complex are not related to different tabanid species but rather to different host species. Further research is needed to shed light on the genetic diversity and vector-parasite specificity of the *T. theileri* species complex in different tabanid species based on partial cathepsin L-like protein (CATL) gene, ITS and Tth625 fragment sequences.

## Figures and Tables

**Table 2:** Prevalence of *Trypanosoma* spp. in Tabanidae of different species. * There are 9 haplotypes in total, as different tabanid species have the same haplotypes.

Species	No. of Positive Isolates (All Isolates)	No. of *Trypanosoma* spp. Sequences (Number of Haplotypes) from Isolates
*Chrysops caecutiens*	0 (1)	
*Chrysops italicus*	0 (1)	
*Chrysops relictus*	0 (3)	
*Haematopota crassicornis*	0 (1)	
*Haematopota pluvialis*	2 (17)	2 (2)
*Haematopota subcylindrica*	1 (14)	1 (1)
*Hybomitra bimaculata*	2 (3)	2 (1)
*Hybomitra distinquenda*	1 (1)	1 (1)
*Hybomitra lapponica*	1 (1)	1 (1)
*Hybomitra lurida*	1 (1)	1(1)
*Hybomitra muehlfeldi*	1 (1)	1 (1)
*Hybomitra nitidifrons*	14 (18)	5 (3)
*Tabanus bromius*	6 (13)	4 (2)
*Tabanus maculicornis*	22 (26)	12 (6)
Total	51 (101)	30 (9) *

## Data Availability

All relevant data are within the manuscript.
